# *Salmonella enterica* Serotype Arizonae Meningitis in a Neonate

**DOI:** 10.1155/2013/813495

**Published:** 2013-09-30

**Authors:** Wubishet Lakew, Abayneh Girma, Elizabeth Triche

**Affiliations:** Department of Pediatrics and Child Health, University of Gondar, 196 Gondar, Ethiopia

## Abstract

Typhoidal and nontyphoidal salmonella infections are common causes of gastroenteritis in the community. However, salmonella only rarely causes invasive infections like meningitis. We report a 13-day-old female neonate with signs and symptoms of meningitis whose cerebrospinal fluid (CSF) culture showed *Salmonella enterica* serotype Arizonae that was sensitive to ceftriaxone. She presented with fever and failure to feed for 2 days. Despite prompt treatment with ampicillin, gentamicin, and ceftriaxone, she developed communicating hydrocephalus, frequent seizures, and coma that progressed to death after 2 weeks of hospitalization. *Salmonella enterica* serotype Arizonae is a rare cause of human infection known to leading to meningitis symptoms similar to those caused by other salmonella species. This is the first report of it as a cause of meningitis in a child under one month of age. Therefore, it should be included in the differential diagnosis of Gram-negative bacillary meningitis in immunocompromised children, neonates, and those with contacts with reptiles.

## 1. Introduction

While typhoidal and non-typhoidal salmonella are common causes of community-acquired infections in developing countries, they rarely cause invasive infections like meningitis in healthy patients. Salmonellae meningitis has been described in newborns, who are more likely to suffer from the disease and more likely to show increased morbidity and mortality as compared with older children and adults. In those who are ill and survive such infections, the chance of complications and relapse is very high despite appropriate antibiotic treatment [1–3]. 

## 2. Case Report

A 13-day-old full-term, previously healthy female patient was brought to the pediatric outpatient department by her mother, who reported the child had fever and failure to feed for two days. The mother denied associated cough, rhinorrhea, emesis, or diarrhea in the patient. The neonate had previously been alert and bottle-fed normally since the time of postnatal hospital discharge. 

At the time of presentation, the patient's exam was notable for axillary temperature of 38.8°C, depressed suck, and absent Moro reflex on neurologic exam. Oxygen saturation was 97% on room air. No medications, including antipyretics, had been given to the patient prior to arrival.

The patient was born by normal spontaneous vaginal delivery to a primigravid 23-year-old college student who had received no antenatal care. She presented to the hospital's maternity ward in active labor at 40-week gestation. She reported an uneventful pregnancy course, denying any significant febrile illness during her pregnancy as well as any history of genital tract lesions, pain, or discharge. 

Spontaneous rupture of membranes occurred 12 hours prior to delivery and subsequent labor and delivery were uncomplicated. The neonate weighed 3000 g and was brought skin-to-skin after a spontaneous cry, requiring no resuscitation. Apgar scores were not reported. 

The patient was admitted to the hospital for medical workup and management. Laboratory investigations upon admission included a complete blood count (CBC), lumbar puncture, liver function tests (LFTs), urinalysis, urine culture, and blood culture. CBC was significant for anemia, with hematocrit of 34.9% and low white blood cell count (WBC) of 3.2 cells/*μ*L (normal range, 5.0–20.0 cells/*μ*L) with a differential showing 48% lymphocytes, 42% neutrophils. Platelets, electrolytes, blood urea nitrogen, creatinine, transaminases, bilirubin, and a urinalysis were within normal limits. Lumbar puncture resulted in cerebrospinal fluid (CSF) that was cloudy in appearance. CSF analysis showed a high WBC count of 1600 cells/*μ*L WBCs (normal range, 0–30 cells/*μ*L) and differential of 96% lymphocytes, 4% neutrophils. CSF protein and glucose were not determined. A Gram stain showed no bacteria. Cerebrospinal fluid culture showed *Salmonella enterica *serotype Arizonae, which was sensitive to ceftriaxone, cefotaxime, chloramphenicol, and ciprofloxacin. Blood and urine cultures showed no growth. 

Of note, the child's mother denied contact with domestic pets or any kind of wild animals including reptiles during and after the pregnancy. 

The patient was initially started on ampicillin, gentamicin, and ceftriaxone after 1 hour of admission. Coverage was narrowed by discontinuation of gentamicin after sensitivities were obtained. Throughout her hospitalization, the patient remained critical, with coma, frequent focal tonic seizures, and alternating episodes of hypothermia and fever, regardless of interventions to achieve thermoregulation. Seizures were characterized by focal tonic and occurred up to 2–4 times a day, in spite of phenobarbitone treatment. Blood glucose and serum electrolytes, including serum calcium measurement, were within normal limits. A cranial ultrasound done on the 20th day of life showed communicating hydrocephalus. After progressive deterioration of mental status throughout hospital, the patient died on her 27th day of life. 

## 3. Discussion

Salmonella organisms are Gram-negative, flagellated facultatively anaerobic bacilli which commonly cause food-borne illnesses. In Africa, non-typhoidal salmonellae are common causes of bloodstream infections in children younger than five years, with symptoms ranging from gastroenteritis to enteric fever [4–7]. 

Invasive salmonellosis in neonates is very rare, accounting for a very small proportion of all bacterial meningitis in neonates. The salmonella serotypes most often reported to cause neonatal meningitis include *S. typhimurium*, *S. paratyphi* B, and *S. typhi* [8]. 

*Salmonella enterica* serotype Arizonae, the organism isolated from this patient's CSF, is a Gram-negative bacillus and a member of the family Enterobacteriaceae. It is biochemically distinguished by its ability to ferment lactose, utilize malonate, and liquefy gelatin and its inability to grow in the presence of KCN. The identification of H_2_S, known to be produced by the organism, in laboratory sample fluids is an important diagnostic clue for routine screening [9, 10]. 

*Salmonella enterica* serotype Arizona (*S. enterica *subspecies IIIa) is naturally found in reptiles but is a known cause of occasional outbreaks of salmonellosis in turkeys and sheep. It has not commonly been described as a cause of disease in immunocompetent individuals in the literature to date, but there are sporadic reports of enteritis and serious disseminated disease in humans. 

The first human infection of *Salmonella enterica* serotype arizonae was reported in 1939 by Caldwell et al. [11]. A literature review done in 2003 by Mahajan et al. showed 17 documented cases of human infection by this organism, of which 11 were children and 4 were infants. All of these infections were traced back to contacts with reptiles like snakes. Patients reported to have *S. enterica* in the literature have generally been older children and adults suffering from otitis media, meningitis, osteomyelitis, pleural effusion, and sinusitis. There is no report of meningitis in the first 28 days of life due to this organism in the literature, which makes this case the first reported case of meningitis caused by this organism to date [12].

Human infection by *Salmonella enterica *serotype Arizonae commonly occurs in individuals with underlying disease or immunodeficiency but has also reportedly afflicted infants infected by intimate contacts with reptile pets. Neonates are known to be relatively immunodeficient, due to lack of fetal antigenic exposure. This may be the reason we have such an unusual infection in our case. The most common manifestation of infection with *S. enterica* is a gastroenteritis similar to that found in other salmonelloses. This occurs in 73% of those infected within the first 3 months of exposure. Our patient, however, showed no signs of gastroenteritis [13]. 

Our patient was treated empirically with ampicillin, gentamicin, and ceftriaxone, based on our experience in treating gram-negative bacillary meningitis, but there was no sign of improvement. Treatment of salmonella meningitis is known to be difficult. While several studies have reported use of different types of antibiotics like cephalosporins, ampicillin, gentamicin, and fluoroquinolones, no standard efficacious treatment has emerged. Though many authorities recommend the use of third-generation cephalosporins as good treatment option, treatment is best determined by organism sensitivities to specific antibiotics obtained through culture [14, 15]. 

Prognosis of salmonella meningitis is very poor. Many neonates and infants die or survive with complications like hydrocephalus, seizures, sensorineural hearing loss, and developmental abnormalities [3]. Like children with salmonella meningitis on appropriate antibiotic treatment in other case reports, our patient had uncontrolled seizures and hydrocephalus despite aggressive treatment.

## 4. Conclusion

*Salmonella enterica* serotype Arizonae is a rare cause of human infection. It can cause meningitis with manifestations similar to meningitis caused by other salmonella species. It should be included in the differential diagnosis of Gram-negative bacillary meningitis, especially in immunocompromised children, neonates, and those with contacts with reptiles. 
